# Anti-stress neuropharmacological mechanisms and targets for addiction treatment: A translational framework

**DOI:** 10.1016/j.ynstr.2018.08.003

**Published:** 2018-08-11

**Authors:** Mark K. Greenwald

**Affiliations:** Department of Psychiatry and Behavioral Neurosciences, School of Medicine, Department of Pharmacy Practice, Eugene Applebaum College of Pharmacy and Health Sciences, Wayne State University, Detroit, MI, 48201, USA

**Keywords:** Stress, Addiction, Drug seeking, Self-administration, Mechanisms, Medications

## Abstract

Stress-related substance use is a major challenge for treating substance use disorders. This selective review focuses on emerging pharmacotherapies with potential for reducing stress-potentiated seeking and consumption of nicotine, alcohol, marijuana, cocaine, and opioids (i.e., key phenotypes for the most commonly abused substances). I evaluate neuropharmacological mechanisms in experimental models of drug-maintenance and relapse, which translate more readily to individuals presenting for treatment (who have initiated and progressed). An affective/motivational systems model (three dimensions: valence, arousal, control) is mapped onto a systems biology of addiction approach for addressing this problem. Based on quality of evidence to date, promising first-tier neurochemical receptor targets include: noradrenergic (α1 and β antagonist, α2 agonist), *kappa*-opioid antagonist, nociceptin antagonist, orexin-1 antagonist, and endocannabinoid modulation (e.g., cannabidiol, FAAH inhibition); second-tier candidates may include corticotropin releasing factor-1 antagonists, serotonergic agents (e.g., 5-HT reuptake inhibitors, 5-HT3 antagonists), glutamatergic agents (e.g., mGluR2/3 agonist/positive allosteric modulator, mGluR5 antagonist/negative allosteric modulator), GABA-promoters (e.g., pregabalin, tiagabine), vasopressin 1b antagonist, NK-1 antagonist, and PPAR-γ agonist (e.g., pioglitazone). To address affective/motivational mechanisms of stress-related substance use, it may be advisable to combine agents with actions at complementary targets for greater efficacy but systematic studies are lacking except for interactions with the noradrenergic system. I note clinically-relevant factors that could mediate/moderate the efficacy of anti-stress therapeutics and identify research gaps that should be pursued. Finally, progress in developing anti-stress medications will depend on use of reliable CNS biomarkers to validate exposure-response relationships.

## Highlights

• Selective review of stress-potentiated seeking/use of nicotine, alcohol, opioids, cocaine, and cannabis.
• Affective/motivational theoretical model is mapped onto a systems biology approach.
• Neurochemical targets are discussed, and potential combination approaches are identified.
• Clinically-relevant factors that could influence efficacy of anti-stress therapeutics are discussed.
• Research gaps are identified and future avenues of investigation are suggested.

## 1 Introduction

### 1.1 Scope of review

Stress-related substance use poses a critical challenge for treating all substance use disorders (SUDs), yet this scientific field is at an early investigational stage. Candidate therapies for attenuating stress-potentiated drug-seeking/use include medications, neuro-stimulation, cognitive-behavioral, exercise/physical activity, and other approaches. Due to that panoramic breadth, this selective review focuses on neuropharmacological strategies grounded in preclinical and clinical evidence that offer translational promise for reducing stress-induced seeking and consumption of nicotine, alcohol, marijuana, cocaine, and opioids. This review does not discuss effects of medications on attenuating drug-abstinence signs/symptoms (which are stressful); although withdrawal effects can lead to drug seeking, that outcome is not inevitable, mostly relates to physical dependence on specific substances, and retaining that topic would greatly lengthen this review. In short, this restricted scope highlights neurochemical mechanisms for reducing stress-induced seeking/self-administration for the most prevalent abused substances. Literature gaps are identified as a means to advance this field of inquiry.

### 1.2 Neurobehavioral mechanisms underlying drug-seeking/self-administration

Mechanisms that link stress reactivity to psychoactive substance use/abuse are complex and incompletely understood. Stressors modulate the neurobehavioral effects of abused substances in stressor-, procedure- and drug-class-specific ways, but also activate some common brain pathways (Armario, 2010; Lopez et al., 1999). Excellent reviews at a more granular level than this translational review indicate numerous neurochemical systems modulate stress-induced substance-seeking/use including noradrenaline (NA), hypothalamic-pituitary-adrenal (HPA) axis including corticotrophin-releasing factor (CRF) and glucocorticoids, opioid, endocannabinoid (eCB), serotonin (5-HT), orexin/hypocretin, dopamine (DA), glutamate, and γ-amino-butyric acid (GABA) (Briand and Blendy, 2010; Dunn and Swiergiel, 2008; Groeneweg et al., 2011; Herman and Cullinan, 1997; Koob, 2010; Mantsch et al., 2016; Plaza-Zabala et al., 2012; Shalev et al., 2000, 2010; Sinha, 2008; Sinha et al., 2011; Viveros et al., 2005), and other systems that are less well studied (see section 2.10). Due to this “neuro-symphony of stress” (Jöels and Baram, 2009), it is only feasible to study a limited number of mechanisms at the same time. When relevant, I mention research that has examined pairwise interactions of transmitter systems.

**Fig. 1:**
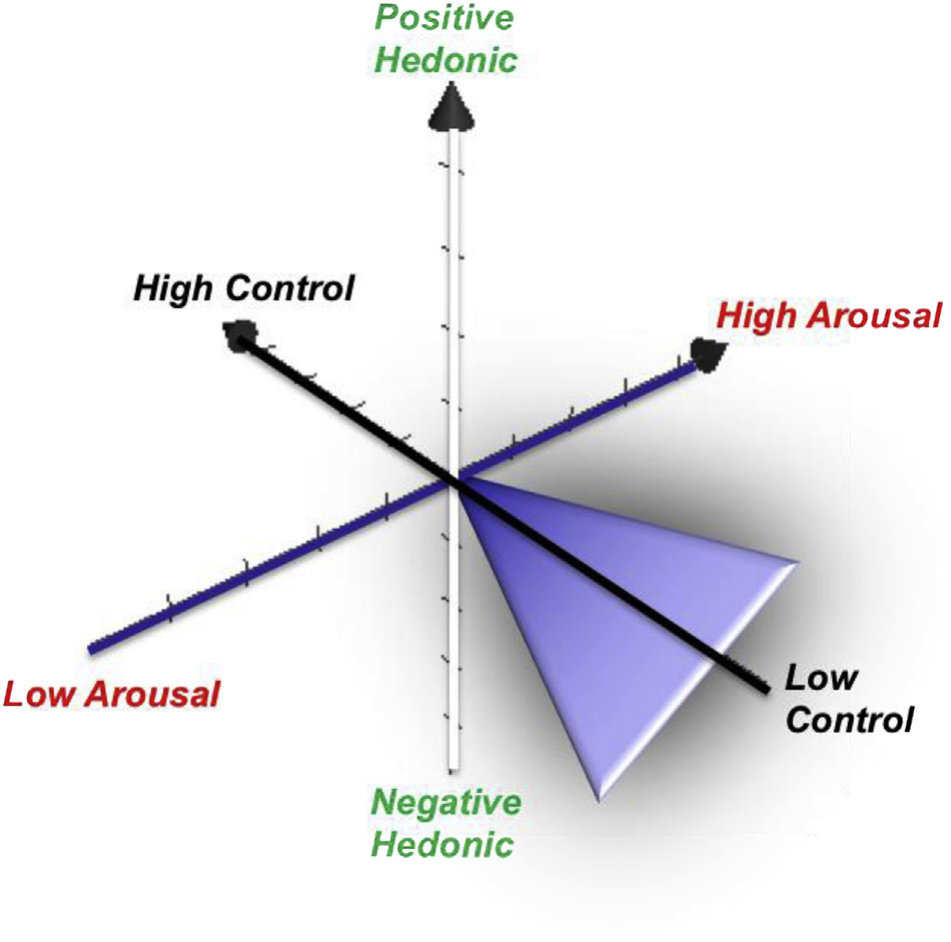
Motivational Systems: Stress-induced substance use behaviors are a function of three motivational dimensions: hedonic valence (approach/avoidance), arousal/activation, and self-control (inhibition/disinhibition). Cone depicts the motivational sector (negative hedonic, high activation, and disinhibition) in which stressors are predicted to amplify drug seeking.

A *motivational systems model* (Fig. 1), based on affective neuroscience theories (Alcaro and Panksepp, 2011; Baker et al., 2004; Davidson et al., 2000; Diekhof et al., 2008) suggests that three empirically separable dimensions may underlie stress-induced drug seeking/use, being greatest at the nexus of negative-hedonic or dysphoric (avoidance-punishment), high-arousal (activation), and low-control (disinhibition) states. Based on research in the *systems biology of addiction* (Aston-Jones and Harris, 2004; Briand and Blendy, 2010; Hester and Garavan, 2004; Koob, 2008; Shalev et al., 2000; Sinha, 2008; Spanagel et al., 1992; Waselus et al., 2011; Zhou et al., 2010), this review adopts the approach that stress-related drug-seeking/use is a function of dysregulated neural (particularly limbic) systems underlying these affective/motivational dimensions. Throughout this review, I link candidate anti-stress pharmacological approaches to these motivational dimensions (to the extent that current evidence allows).

### 1.3 Experimental models of stress-induced drug-seeking/use

Experimental approaches to studying stress-related drug-seeking/use can be classified with regard to: (a) type of stressor, e.g., physical, environmental, and pharmacological, (b) stage in the behavioral cycle of addiction (initiation, progression, maintenance, relapse), and (c) drug-seeking outcome measure (e.g., operant responding for drug, conditioned place preference). This literature review focuses on models of *maintenance and relapse stages*, which more closely translate to individuals presenting for SUD treatment (who have already initiated and progressed). Most studies with laboratory animals to date have measured reinstatement of operant responding for drug as the outcome measure; fewer studies have measured conditioned place preference (CPP) but these are included in this review for comprehensiveness. The CPP model has limitations, particularly because drug is experimenter-administered and drug exposure is minimal.

As summarized in Table 1, drug seeking/consumption is enhanced by a wide range of stressors including: social factors (defeat, isolation), physical factors (restraint, cold swim, food deprivation, footshock), and pharmacological probes (e.g., *α*2-adrenoceptor antagonist yohimbine [section 2.1], *kappa*-opioid agonist, and neuropeptides [sections 2.10, 2.2.1, 2.7]). Each stressor type has strengths and weaknesses, and translation of these models to clinical treatment is limited by several moderating factors, briefly discussed next.

**Table 1:** Studies demonstrating a significant direct effect of stressor on reinstatement of (or increase in) seeking behavior, by type of abused substance.

Stress Method	Nicotine	Alcohol	Cocaine	Opioid
**Chemical stressors**				
Yohimbine	Costello et al., 2014; Feltenstein et al., 2012; Grella et al., 2014; Gueye et al., 2016; Nygard et al., 2016; Woodcock et al., under review	Ayanwuyi et al., 2013; Aziz et al., 2016; Baarendse et al., 2014; Bertholomey et al., 2013, 2016; Ciccocioppo et al., 2014; Cippitelli et al., 2008, 2010, 2015; Funk et al., 2014, 2016; Gass and Olive, 2007; Gonzalez-Cuevas et al., 2018; Haack et al., 2014; Kastman et al., 2016; Lê et al., 2005, 2009, 2011, 2013; Marinelli et al., 2007; Nielsen et al., 2012; Richards et al., 2008, 2009; Rorick-Kehn et al., 2016; Ryan et al., 2013; Schank et al., 2014, 2015; Sheth et al., 2017; Simms et al., 2011, 2012; Stopponi et al., 2011, 2012, 2013	Ahmed and Koob, 1997; Anker and Carroll, 2010; Brown et al., 2012; Chauvet et al., 2009, 2014, 2016; de Guglielmo et al., 2013; Feltenstein and See, 2006; Feltenstein et al., 2011; Fletcher et al., 2008; Gonzalez-Cuevas et al., 2018; Lee et al., 2004; Kupferschmidt et al., 2009; Mantsch et al., 2010,^a^; Regier et al., 2014; Schank et al., 2014; Schmeichel et al., 2017; Schmoutz et al., 2012; Schroeder et al., 2013; Williams et al., 2014; Williams et al., 2016; Zhou et al., 2012	de Guglielmo et al., 2017; Greenwald et al., 2013^b^; Stopponi et al., 2014; Zhou et al., 2013
Noradrenaline			Brown et al., 2009, 2012	
CRF		Lê et al., 2002	Blacktop et al., 2011, 2016; Brown et al., 2009; Buffalari et al., 2012; Graf et al., 2011; Kupferschmidt et al., 2012; Mantsch et al., 2008	Shaham et al., 1997
Caffeine			Regier et al., 2014; Swalve et al., 2016	
Kappa-agonist	Grella et al., 2014	Funk et al., 2014; Lê et al., 2018	Al-Hasani et al., 2013a,^a^, 2013b,^a^; Land et al., 2009,^a^; Redila and Chavkin, 2008; McLaughlin et al., 2006a,^a^; Valdez et al., 2007	
Orexin-1	Plaza-Zabala et al., 2010		Boutrel et al., 2005; Harris et al., 2005	Harris et al., 2005
Neuropeptide S		Cannella et al., 2009	Kallupi et al., 2010; Paneda et al., 2009	
**Non-chemical stressors**				
Intermittent electric footshock	Buczek et al., 1999; Bruijnzeel et al., 2009, 2010; Plaza-Zabala et al., 2010; Yamada and Bruijnzeel, 2011; Zislis et al., 2007	Cippitelli et al., 2011; de Guglielmo et al., 2014; Economidou et al., 2006, 2007; Hansson et al., 2006; Lê et al., 1998, 1999, 2000, 2002, 2005, 2006; Liu and Weiss, 2002; Martin-Fardon et al., 2000; Schank et al., 2012; Sidhpura et al., 2010; Zhao et al., 2006	Ahmed et al., 2000; Beardsley et al., 2005, 2010; Erb et al., 1996, 1998, 2000; Graf et al., 2011; Kupferschmidt et al., 2009, 2012; Mantsch and Goeders, 1999; Mantsch et al., 2008, 2010,a; Martin-Fardon et al., 2000; Martin-Fardon and Weiss, 2012; Redila and Chavkin, 2008; Vranjkovic et al., 2014; Wang et al., 2007, 2009	Kuzmin et al., 1996; Qi et al., 2013,^a^; Shaham et al., 1993, 1996, 2000b; Shaham and Stewart 1994, 1995, 1996; Shalev et al., 2000, 2001; Wang et al., 2006,^a^; Zhou et al., 2008, 2015
Social defeat		Funk et al., 2005; Molander et al., 2012; Riga et al., 2014	Land et al., 2009,^a^; Manvich et al., 2016	Ribeiro Do Couto et al., 2006,^a^
Social isolation			Bozarth et al., 1989	Alexander et al., 1978; Bozarth et al., 1989
Physical restraint		Walker et al., 2015	Tung et al., 2016	Meng et al., 2014,^a^; Ribeiro Do Couto et al., 2006,^a^
Forced swim	Jackson et al., 2013,^a^		Carey et al., 2007; Conrad et al., 2010; Graziane et al., 2013; McLaughlin et al., 2006b,^a^; McReynolds et al., 2014,^a^; Pastor et al., 2011; Polter et al., 2014; Schindler et al., 2012; Vranjkovic et al., 2012,^a^	Karimi et al., 2014,^a^; Zanos et al., 2014
Food deprivation			Carroll and Meisch, 1984; Chen et al., 2014	Sedki et al., 2015; Shalev et al., 2000, 2006; Tobin et al., 2013

a CPP paradigm.

b Clinical study.

### 1.4 Translation of experimental models to clinical application: moderator variables

Experimental models of stress-related drug seeking/use have not routinely included clinically-relevant factors that might mediate/moderate the efficacy of anti-stress therapeutic agents. Next, I briefly discuss selected factors worthy of investigation in developing anti-stress pharmacotherapies in these models. Detailed analysis of these factors exceeds the scope of this review, but the purpose is to prime the reader to consider these factors in the context of the discussion that follows in section 2.

#### 1.4.1 History of substance exposure/use

The extent to which acute stressors augment the reinforcing effects of psychoactive substances may partly depend on the subject's history of substance exposure (e.g., *in utero*) or use (i.e., voluntary intake). Chronic and/or escalating substance intake has been shown to produce stress-like responses that, in turn, sensitize animals – via CRF, glucocorticoid, and glutamatergic mechanisms – to acute stress-potentiation of the reinforcing effects of cocaine (Ahmed et al., 2000; Graf et al., 2011; Mantsch et al., 2008; Sorge and Stewart, 2005) and alcohol (Liu and Weiss, 2002; Sidhpura et al., 2010). These data suggest that agents capable of desensitizing these neurochemical systems could be therapeutically useful, especially for subjects with extensive chronic exposure or high levels of tolerance/dependence.

#### 1.4.2 Environmental factors

Research into effects of stressors on drug-motivated behavior has not adequately investigated the environmental context, particularly non-drug choice alternatives. Higher magnitude or probability of non-drug positive reinforcers in the environment during stress-exposure may bias animals away from habitual drug seeking, i.e., serve a stress-protective function. This phenomenon has been modeled in rats using environmental enrichment (Chauvet et al., 2009). Future studies should examine whether anti-stress pharmacotherapies are more effective in combination with non-drug positive reinforcers. In contrast, economic insecurities among humans (i.e., dearth of positive reinforcement in a person's environment) are chronic stressors that contribute to substance use. Furthermore, magnitude and/or probability of punishers in the subject's environment may play an important role in modulating stress-induced drug seeking, although this has rarely been investigated. For instance, early social isolation of rats enhances cocaine motivation, but isolation did not affect extinction or stress-reinstatement of cocaine seeking (Baarendse et al., 2014). Future studies should investigate whether drug intake that is suppressed by punishment (cf. Pelloux et al., 2007) is more easily reinstated by stress than drug intake that is suppressed by non-drug positive reinforcers, and whether other factors such as drug history moderate these effects (see Smith and Laiks, 2017).

#### 1.4.3 Sleep loss

Sleep loss (and/or circadian disruption) is a biological stressor but has rarely been investigated in studies of drug seeking/self-administration. This is noteworthy because insomnia is a predictor of relapse to substance abuse (Roehrs and Roth, 2015; Kaplan et al., 2014). Also, sleep loss causes hyperalgesia (Alexandre et al., 2017; Roehrs et al., 2006; Vanini, 2016) that, in turn, may increase misuse of opioids and other substances (Edwards et al., 2011; Hipólito et al., 2015; Wachholtz et al., 2015). Recognizing the role of insomnia as a stressor leads to the idea of treating insomnia as one cause of substance use. Non-addictive sleep-promoting agents might be useful in this regard (see section 2.7).

#### 1.4.4 Chronic pain

Stress can increase nociceptive signaling and contribute to chronic pain states (Ghitza, 2016; Imbe et al., 2006; Jennings et al., 2014; Johnson and Greenwood-Van Meerveld, 2014). This overlap is important because it suggests (1) stress-exposure could cross-sensitize subjects – presumably via shared neurobiological pathways – to drug seeking, and (2) therapeutics used to attenuate stress-reactivity could have dual actions on analgesia and drug seeking/use. On the other hand, it is well established that stress exposure can sometimes produce analgesia (Butler and Finn, 2009; Parikh et al., 2011). Thus, parametric studies are needed to examine basic interactions between (acute and chronic) stressors and (acute and chronic) nociceptive stimulation, and to translate this knowledge toward the development of therapeutic approaches that could potentially address both conditions.

#### 1.4.5 Chronic stress and mental health conditions

Early-life or chronic stress as well as psychiatric conditions (e.g., anxiety, depression) are highly prevalent among individuals with SUDs and may increase risk of relapse to substance use especially under stressful conditions (Chida and Hamer, 2008; Lijffijt et al., 2014). Experimental animal models have only infrequently created such a behavioral history (e.g., repeatedly exposing animals to anxiety-provoking or helplessness-provoking conditions) or exposed adolescent animals to the biological stressor corticosterone (Al-Hasani et al., 2013a; Bertholomey et al., 2016) to determine its effects on stress-induced drug seeking/use. Further systematic research would be valuable for understanding a complex yet important set of clinically relevant influences.

#### 1.4.6 Trait factors

Impulsivity is an example of one trait variable that may influence stress-induced drug seeking behavior; furthermore, this trait maps onto a key motivational dimension, disinhibition/control, proposed in this review (Fig. 1). Specifically, high trait impulsivity (associated with less prefrontal cortical inhibitory control) could, in the presence of a stressor, result in less resistance to drugs and related cues, making the individual more prone to substance use. We have found that the relationship between trait impulsivity on current mood state (e.g., Beck Depression Inventory scores) is mediated by effects of cumulative adverse drug-use consequences for both cocaine and heroin users (Lister et al., 2015; Reid et al., 2018). Other inter-related trait variables such as anxiety sensitivity (Lejuez et al., 2008), distress tolerance (Brown et al., 2005), emotion regulation (Naragon-Gainey et al., 2017), and resilience or coping ability (Belding et al., 1996; Hyman et al., 2009) are stress-mediator/moderator variables that are not routinely assessed in human studies, but these might potentially predict drug abstinence during addiction treatment (Strong et al., 2012).

#### 1.4.7 Biological sex

Chronic manipulation of HPA axis (CORT) during adolescence increases sensitivity to YOH-induced alcohol seeking in female more than male adult rats (Bertholomey et al., 2016). Neonatal isolation stress increased cocaine seeking behavior in adult rats, however, effects did not differ for males and females (Lynch et al., 2005). Some effects of acute stressors on relapse-like behavior are sex-dependent, with females typically more sensitive especially during high-estradiol levels (Anker and Carroll, 2010; Back et al., 2005; Chartoff and Mavrikaki, 2015; Feltenstein et al., 2011; Swalve et al., 2016; Verplaetse et al., 2015) although not all studies observe clear sex differences in stress-induced drug seeking (Buffalari et al., 2012; Zhou et al., 2012; also, see reviews by Becker et al., 2012; Bobzean et al., 2014; Fox and Sinha, 2009; Hudson and Stamp, 2011). Consistent with current NIH policy, studies should be adequately powered to detect sex differences in stress-induced drug seeking/use as well as candidate pharmacotherapies that may attenuate these effects.

#### 1.4.8 Neurotransmission-related genetic factors

Few studies have addressed the role of genetic variation in stress-related substance use and relapse, in contrast to genetic association studies of initial addiction vulnerability. We reported that, in heroin-dependent volunteers undergoing buprenorphine dose tapering with an abstinence incentive, variations in genes encoding the *kappa*-receptor (*OPRK1* rs3802281; Greenwald et al., 2012) and glucocorticoid receptor (*NR3C1* rs6877893; Greenwald and Burmeister, 2018) predicted opioid relapse potential. Variation in *OPRK1* rs6989250 is also associated with risk of cocaine relapse (Xu et al., 2013). Although CRH-binding protein (*CRHBP*) genotype variation moderated the association between stress-induced negative affect and negative consequences of alcohol intake in heavy-drinking subjects (Tartter and Ray, 2012), effects of this genotype on stress-induced drinking were not studied. Nonetheless, CRF is a promising target, as *CRHR1* knockout animals are less sensitive to stress-induced alcohol intake (Hansson et al., 2006; Molander et al., 2012; Pastor et al., 2011). CRF-R1 knockdown mice are also less sensitive to stress-reinstatement of cocaine seeking (Chen et al., 2014).

## 2 Neuropharmacological targets

This section reviews evidence from studies related to various neurochemical systems that offer anti-stress therapeutic potential. To promote translational studies, each section indicates positron emission tomography (PET) imaging radiotracers that could be used to investigate proof-of-targeting in future prospective studies.

### 2.1 Noradrenergic system

The NA system has been the most commonly studied neurochemical domain for stress-related substance use, alone or in combination with other systems (see below). Discontinuation of chronic exposure to nicotine (Bruijnzeel et al., 2010; Sofuoglu et al., 2003), alcohol (Muzyk et al., 2011), cocaine (McDougle et al., 1994; Sofuoglu and Sewell, 2009), and opioids (Maldonado, 1997; Van Bockstaele et al., 2001) is a functional stressor associated with increased NA neurotransmission. It has been hypothesized that elevated NA release in the extended amygdala, and altered DA-mediated plasticity in the ventral tegmental area (VTA), alter hedonic processing of drug-related stimuli and are common substrates in withdrawal-associated relapse to drug seeking (Aston-Jones and Harris, 2004; Espana et al., 2016; Fitzgerald, 2013; Smith and Aston-Jones, 2008; Weinshenker and Schroeder, 2007).

Yohimbine (YOH) is an α_2_-adrenoceptor antagonist that increases NA neurotransmission by blocking feedback at presynaptic autoreceptors (Doxey et al., 1984; Goldberg and Robertson, 1983) and has become an important tool for investigating stress-related drug seeking/use. YOH-mediated increases in NA release and synaptic levels regulate HPA axis activity (Armario, 2010; Banihashemi and Rinaman, 2006; Grunhaus et al., 1989; Leri et al., 2002; Smythe et al., 1983), as well as 5-HT and DA neurotransmission (Brannan et al., 1991; Cheng et al., 1993; Hopwood and Stamford, 2001; Maura et al., 1982; McCall et al., 1991; Millan et al., 2000; Mongeau et al., 1993; Raiteri et al., 1990; Söderpalm et al., 1995a, b; Winter and Rabin, 1992). In a PET neuroimaging study of rhesus monkeys, YOH increased [^11^C]-flumazenil binding potential (Matsunaga et al., 2001) indicating YOH actions at GABA-A receptors that might correlate with its anxiogenic (negative-hedonic, arousing) and/or disinhibiting motivational effects (Fig. 1).

YOH has been used extensively as an experimental stressor in animal and human laboratory models. It produces anxiogenic effects in animals, healthy subjects, patients with panic disorder and opioid use disorder, which can be blocked by the α2-adrenoceptor agonist clonidine (Albus et al., 1992; Bremner et al., 1996; Cameron et al., 2000; Charney et al., 1983, 1992; Gurguis et al., 1997; Mattila et al., 1988; Pellow et al., 1987; Stine et al., 2002). These anxiogenic effects are presumed to mediate the effects of YOH on the reinforcing effects of drugs and drug-related stimuli. Reviews have concluded that YOH is a reliable and potent inducer of drug seeking with translational value (Bossert et al., 2005; Figlewicz et al., 2014; See and Waters, 2011; Shaham et al., 2000a). On the other hand, Chen et al. (2015) found that the effect of YOH on food-reinforced operant behavior may partly depend on factors unrelated to stress-induction; namely, YOH did not induce conditioned place aversion, YOH increased responding independent of the rat's history of contingent self-administration of food reward, and YOH increased medial prefrontal cortex (but not nucleus accumbens) dopamine levels. As a result, Chen et al. proposed that YOH may invigorate responding for cues that possess weaker rewarding effects in rodents (also see Box 2 in Mantsch et al., 2016). Given the extensive use of YOH in experimental stress studies, such findings warrant caution in relying only on YOH as an experimental model, yet encourage further investigation to determine the precise conditions under which YOH exerts its response-enhancing effects. For instance, YOH effects could differ for food vs. drug reinforcers, for paradigms involving drug-paired cues, for single-operant vs. choice scenarios, the subject's behavioral history, or other methodological factors.

In animal studies, YOH administration delays extinction of cocaine seeking (Kupferschmidt et al., 2009) and – as summarized in Table 1 – YOH robustly reinstates previously extinguished responding for nicotine, alcohol, cocaine, and opioids. Reinstatement of alcohol seeking by YOH (previously established to be a 5-HT1A partial agonist; Millan et al., 2000; Winter and Rabin, 1992) was blocked not only by the α2 agonist clonidine but also by a selective 5-HT-1A antagonist (WAY-100,635), suggesting an important role for 5-HT in mediating YOH-induced alcohol seeking (Lê et al., 2009; see section 2.3). Although YOH did not increase heroin seeking in opioid-dependent rats (Minhas and Leri, 2014), our group was the first to find that YOH increased drug seeking in humans, i.e., the stressor increased opioid-maintained responding in a sample of buprenorphine-maintained heroin-dependent human subjects (Greenwald et al., 2013) and we recently extended this to cigarette puff-maintained responding in tobacco users (Woodcock et al., under review). Tests of YOH to reinstate Δ^9−^THC- responding have not been reported (due to limitations of animal models in demonstrating reinforcing effects of Δ^9^-THC), although chronic Δ^9−^THC exposure during adolescence augmented YOH reinstatement of heroin seeking (Stopponi et al., 2014).

Furthermore, YOH augments cue-induced reinstatement for nicotine (Costello et al., 2014), heroin (Banna et al., 2010) and cocaine (Buffalari and See, 2011; Feltenstein and See, 2006; Fletcher et al., 2008) in animals, and enhances cocaine cue-reactivity in cocaine users (Moran-Santa Maria et al., 2014), suggesting this primarily NA stressor can enhance the conditioned reinforcing properties of drug cues to elevate relapse risk. Moreover, across animal and human studies (Adams et al., 2017; Bari and Robbins, 2013; Ma et al., 2003; Sanger, 1988; Schippers et al., 2016; Sun et al., 2010; Swann et al., 2005), YOH and other α2-antagonists increase impulsive responding (i.e., decrease self-control; see Fig. 1), suggesting another mechanism by which YOH could increase substance use (see Table 2). Interestingly, NA binding to α2 receptors in prefrontal cortex regulates attentional set, response inhibition and behavioral flexibility (Arnsten, 1998; Aston-Jones and Cohen, 2005; De Martino et al., 2008; Ramos and Arnsten, 2007; Robbins and Roberts, 2007). YOH also causes perseverative responding (Caetano et al., 2013), which predicts that YOH should promote drug-maintained responding particularly habitual substance use.

**Table 2:** Candidate therapeutic targets, neuropharmacological agents, and related motivational mechanisms for attenuating stress-induced drug seeking/self-administration.

		Stress-motivational dimension^a^			Substance			
CNS Target	Examples	Dysphoria	Arousal	Disinhibition	Nicotine	Alcohol	Cocaine	Opioids
α2-adrenergic agonist	Clonidine, lofexidine, guanfacine	?	+	+	McKee et al., 2015^c^; Yamada and Bruijnzeel, 2011	Lê et al., 2005, 2011	Erb et al., 2000; Lee et al., 2004	Kowalczyk et al., 2015^c^; Shaham et al., 2000b
α1-adrenergic antagonist	Prazosin, doxazosin		+	+		Funk et al., 2016; Haass-Koffler et al., 2017^c^; Lê et al., 2011; Riga et al., 2014; Wilcox et al., 2018^c^		
β-adrenergic antagonist	Propanolol		+				Mantsch et al., 2010,^b^	
CRF-1 antagonist	Antalarmin	+	+		Bruijnzeel et al., 2009; Plaza-Zabala et al., 2010	Lê et al., 2000; Marinelli et al., 2007	Shaham et al., 1998; McReynolds et al., 2014,^b^	Shaham et al., 1998
Glucocorticoid antagonist	Mifepristone		+			Simms et al., 2012; Vendruscolo et al., 2015^c^		Karimi et al., 2014,^b^
*Kappa*-opioid antagonist	Nor-BNI, CERC-501, Arodyn	+	+	?	Grella et al., 2014; Jackson et al., 2013,^b^	Domi et al., 2018; Funk et al., 2014; Lê et al., 2018; Rorick-Kehn et al., 2014; Schank et al., 2012; Sperling et al., 2010,^b^	Al-Hasani et al., 2013b,^b^; Beardsley et al., 2005, 2010; Carey et al., 2007,^b^; Graziane et al., 2013; Redila and Chavkin, 2008	Sedki et al., 2015; Zhou et al., 2013
*Delta*-opioid antagonist	SoRI-9409	+		?		Nielsen et al., 2012		
Nociceptin antagonist	LY2940094	+	+	?		Rorick-Kehn et al., 2016		
eCB (anandamide) enhancer	URB597, Cannabidiol	+	+	?		Gonzalez-Cuevas et al., 2018	Chauvet et al., 2014; Hamilton et al., 2018; Gonzalez-Cuevas et al., 2018	
5-HT-3 antagonist	Ondansetron, tropisetron	+	?	?		Lê et al., 2008		
GABA promoters	Pregabalin, tiagabine		+	?/–		Stopponi et al., 2012	de Guglielmo et al., 2013	
GABA-B agonist	Baclofen	?	+	?		Williams et al., 2016		Meng et al., 2014,^b^
mGluR2/3 agonist	LY379268	+	?	?		Sidhpura et al., 2010; Zhao et al., 2006	Martin-Fardon and Weiss, 2012	
mGluR5 antagonist	MTEP, Fenobam	+				Sidhpura et al., 2010; Zhao et al., 2006	Martin-Fardon and Weiss, 2012	
Orexin-1 antagonist	Suvorexant, SB-334867	+	+	?	Plaza-Zabala et al., 2010	Cannella et al., 2009; Richards et al., 2008; Ubaldi et al., 2016	Boutrel et al., 2005; Kallupi et al., 2010; Paneda et al., 2009; Schmeichel et al., 2017; Wang et al., 2009	Qi et al., 2013,^b^
Oxytocin agonist	Carbetocin	+	?	+				Zanos et al., 2014
Vasopressin 1b agonist	ABT-436	+	+	?				Zhou et al., 2008, 2015
NPS antagonist	RTI-118	+	+				Schmoutz et al., 2012	
NK-1 antagonist	Aprepitant, L822429	?	?			Schank et al., 2011, 2014, 2015	Schank et al., 2014	
Relaxin-3/RXFP3 antagonist	R3(B1-22)R, R3(BΔ23-27)R/I5	+	+			Ryan et al., 2013; Walker et al., 2015		
PPAR-γ agonist	Pioglitazone	?				de Guglielmo et al., 2017		
α3β4 nAChR partial agonist	AT-1001				Yuan et al., 2017	Cippitelli et al., 2015		

a Based on broad, non-exhaustive assessment of empirical literature (using serial PubMed searches that crossed each CNS target label [or closely related terms, e.g., “anxiety” instead of “dysphoria”, “impulsivity” instead of “disinhibition”] with each stress-motivational dimension label in this table, as well as studies reviewed herein), the author's subjective designation of “+” indicates that the CNS target is *likely involved* in counteracting the stress-induced motivation-dimensional outcome. Designation of “?” indicates *mixed evidence or uncertainty*, and blank cells indicate *minimal or no supporting evidence*. The designation of “–“ indicates possible worsening of this motivational feature (particularly with chronic dosing). This global summary is simply intended for guidance in mapping these theoretical dimensions to neurochemical mechanisms and, more importantly, for designing studies to formally test effects of these CNS targets against motivational phenotypes, ultimately moving toward combinations of medications for pan-efficacy (see Table 3).

b CPP paradigm.

c Clinical study.

The foregoing data suggest agents that reduce NA neurotransmission are first-line candidates as anti-stress medications for at least some SUDs; this proposition has been tested in animal studies. As indicated in Table 2, the α2-agonists clonidine, lofexidine and guanfacine block stress-reinstated responding for nicotine, alcohol, heroin, cocaine, and heroin/cocaine “speedball” (Highfield et al., 2001) at doses that blocked footshock-induced NA release in prefrontal cortex and amygdala. We recently proposed (Woodcock et al., under review) that the occupancy ratio of NA receptors (higher-affinity α2-receptors vs. lower-affinity α1-and β-receptors) may underlie stress-induced drug use. Specifically, we hypothesized that during acute stress-exposure, NA released into the synapse may exceed capacity of higher-affinity α2 receptors, possibly leading to spillover binding at lower-affinity α1-and β-receptors; and, in turn, this altered receptor occupancy ratio could potentiate drug seeking. If this theory were confirmed, it could suggest that α1-and β-receptor antagonists may also attenuate drug seeking, especially at higher stress intensities (when NA transmission is greatest). In fact, the α1 antagonists, prazosin and doxazosin, and the α2 agonist guanfacine blocked several types of stress-reinstatement of alcohol-seeking in rodents (Funk et al., 2016; Lê et al., 2011; Riga et al., 2014), as well as YOH-induced motoric impulsivity (Adams et al., 2017). Stress-reinstatement of cocaine seeking is attenuated by the β-receptor antagonist propranolol (Mantsch et al., 2010) and the NA synthesis inhibitor nepicastat (Schroeder et al., 2013). However, when injected directly into the amygdala central nucleus, the α1-antagonist prazosin and the β1/2-antagonist propanolol did not attenuate footshock-reinstatement of nicotine seeking, whereas the α2 agonist clonidine did (Yamada and Bruijnzeel, 2011), highlighting different effects of site-specific versus systemic drug administration across studies. One study found a double dissociation in NA receptor subtypes mediating rewarding and somatic withdrawal effects during mecamylamine-precipitated nicotine withdrawal: only prazosin attenuated brain reward threshold deficits during nicotine withdrawal, whereas only clonidine and propanolol attenuated somatic signs (Bruijnzeel et al., 2010), suggesting that these NA agents address different motivational mechanisms.

In clinical practice, α2-receptor agonists are typically used as antihypertensive agents and for improving cognitive control in attention-deficit/hyperactivity disorder. Clonidine is a second-line treatment for smoking cessation (Gómez-Coronado et al., 2018) and guanfacine exhibited a promising signal in a pilot-test as a anti-stress/cognitive-control agent in tobacco smokers (McKee et al., 2015). Regarding cannabis use disorder, one study found that lofexidine reduced marijuana relapse-like behavior in a human laboratory model (Haney et al., 2008) but was not generally effective, when combined with oral THC, for reducing outpatient cannabis use (Levin et al., 2016). For cocaine use disorder, during early cocaine abstinence, guanfacine attenuated stress reactivity and craving (Fox et al., 2012) and enhanced inhibitory control (Fox et al., 2015), which might treat dysphoric and impaired self-control dimensions in the drug-motivational space (Fig. 1). Another study found guanfacine did not attenuate stress responses, prompting the authors to question its value for cocaine use disorder (Moran-Santa Moria et al., 2015). Furthermore, these human studies focused on drug craving rather than drug seeking/use, which may limit their clinical utility. Another caveat is that a study in rhesus monkeys found that, in the absence of stress, chronic lofexidine treatment produced a leftward shift in the cocaine self-administration dose-response curve; accordingly, the authors suggested α2 agonist maintenance may not be generally useful for cocaine use disorder (Kohut et al., 2013). In opioid-dependent humans, where YOH produced anxiety and opioid withdrawal-like effects (Greenwald et al., 2013; Oliveto et al., 2003; Stine et al., 2002), α2 agonists suppress opioid withdrawal symptoms (Gowing et al., 2014) and stress-induced craving (Jobes et al., 2011; Sinha et al., 2007; but see Moran-Santa Maria et al., 2015). Although maintenance on clonidine (vs. placebo) significantly lengthened duration of opioid abstinence among methadone-maintained patients, survival curve analysis revealed no significant clonidine vs. placebo group difference in time to opioid relapse, suggesting circumscribed efficacy (Kowalczyk et al., 2015). Some data suggest α-2A receptors primarily mediate anti-stress therapeutic effects of α2 agonists (e.g., guanfacine is a α-2A-specific partial agonist), whereas uncertainty remains regarding the roles of α-2B and α-2C subtypes, as well as whether these effects occur via pre-synaptic autoreceptors or heteromers with non-NA neurons such as glutamate (Shields et al., 2009).

α1 antagonists have been investigated for efficacy in alcohol use disorder, with mixed results. In a recent, placebo-controlled, phase 2 trial, six-week treatment with prazosin (up to 16 mg/day) did not exhibit overall efficacy (Wilcox et al., 2018). Interestingly, pretreatment blood pressure – possibly reflecting chronic stress levels – has been found to moderate the efficacy of prazosin and doxazosin in reducing alcohol intake in two clinical trials (Haass-Koffler et al., 2017; Wilcox et al., 2018), such that these medications reduced alcohol intake to a greater degree among patients with higher pretreatment blood pressure.

This review emphasizes a system biology approach to the influence of stress on substance use. Notably, the NA system has been the most extensively studied on its own and interacting with other neurochemical systems. Accordingly, Table 3 summarizes studies that have experimentally analyzed effects of manipulating NA signaling in combination with other systems, specifically for an effect on stress-related substance use. This table is intended to advance the idea that multi-modal medication (polypharmacy or “cocktail”) approaches may be useful, and perhaps preferable (i.e., safer and more effective than unimodal intervention), for treating stress-related substance use. Table 3 also illustrates the current research landscape: alcohol and cocaine have most often been studied using this systems biology approach, but there are substantial research gaps for other abused drugs. From a clinical perspective, it is notable that the use of other (non-NA) medications in combination with NA agents might minimize incidence of hypotension (i.e., produce a dose-sparing effect for the NA medication), which is the primary side effect of the NA medication class. Finally, prescribing NA medications for substance abusers who have hypertension could help treat this common comorbid condition.

**Table 3:** Studies investigating potential interactions between noradrenergic and other neurochemical systems in stress-related substance use.

Neurochemical System	Nicotine	Alcohol	Cocaine	Opioid
CRF-1		Ayanwuyi et al., 2013; Lê et al., 2013; Marinelli et al., 2007	Brown et al., 2009, 2012; McReynolds et al., 2014,^a^	
CORT		Bertholomey et al., 2016; Simms et al., 2012		Greenwald et al. (in prep.)^b^
Orexin-1		Richards et al., 2008; Kastman et al., 2016	Boutrel et al., 2005; Schmeichel et al., 2017	
5-HT		Lê et al., 2009	Land et al., 2009,^a^	
Kappa-opioid	Grella et al., 2014; Nygard et al., 2016	Funk et al., 2014	Al-Hasani et al., 2013b^a^; Valdez et al., 2007	Zhou et al., 2013
Delta-opioid		Nielsen et al., 2012		
Nociceptin		Rorick-Kehn et al., 2016		
eCB	Gueye et al., 2016	Cippitelli et al., 2008	Vaughn et al., 2012	
GABA-B		Williams et al., 2016		
GABA-A			de Guglielmo et al., 2013	
α3β4 nAChR		Cippitelli et al., 2015; Yuan et al., 2017		
PPAR-γ		Stopponi et al., 2011, 2013		de Guglielmo et al., 2017
Neurokinin-1		Schank et al., 2014	Schank et al., 2014	
Neuropeptide S			Schmoutz et al., 2012	
Neuropeptide Y		Cippitelli et al., 2010		

a CPP paradigm.

b Clinical study.

For proof-of-target studies, PET radiotracers exist for measuring occupancy of α2C receptors ([^11^C]-ORM-13070; Arponen et al., 2014) and noradrenaline transporter ([^11^C]-MENET; Adhikarla et al., 2016), but none are available for α1-or β-adrenergic receptors.

### 2.2 HPA axis

#### 2.2.1 Corticotropin-releasing factor (CRF)

As noted in section 1.3, infusion of CRF (which mimics supra-physiologic endogenous release from the hypothalamus) reinstates previously extinguished drug seeking in animal models. Notably, CRF-1 receptor knockout animals are less sensitive to stress-induced cocaine seeking (Chen et al., 2014). Thus, CRF-1 antagonists have been explored for blocking stress-induced reinstatement. Early studies found that ICV infusion of non-selective CRF-1/2 receptor antagonists decreased footshock reinstatement of responding for heroin (Shaham et al., 1997), cocaine (Erb et al., 1998), alcohol (Lê et al., 2000; Liu and Weiss, 2002) and nicotine (Zislis et al., 2007). As summarized in Table 2, selective CRF-1 antagonism attenuated stress-reinstatement of responding for nicotine, alcohol, cocaine and heroin.

Although these preclinical studies suggest CRF-1 antagonism might be a useful approach for reducing stress-induced drug seeking, this apparent promise must be tempered by contradictory results from recent clinical studies. Two CRF-1 antagonists, pexacerfont and verucerfont, failed to demonstrate anti-craving effects in patients with alcohol use disorder (Kwako et al., 2015b; Schwandt et al., 2016). A third CRF-1 antagonist, GSK561679, failed to show anxiolytic effects in a human laboratory model (Grillon et al., 2015) and lacked efficacy in a clinical trial with posttraumatic stress disorder patients (Dunlop et al., 2017). Given this lack of translational efficacy, considerable caution is warranted for similar congeners in this pharmacological class. Several methodological factors could have led to poor translation (Pomrenze et al., 2017; Shaham and de Wit, 2016); if these are properly addressed, there could still be a path forward for these agents but, in all likelihood, therapeutic application may be narrower than originally hoped (Spierling and Zorrilla, 2017). Alternative study designs may be needed that measure not only direct effects of the medication on substance use, but also intermediate stress-related phenotypes, e.g., coping or resilience (Contoreggi et al., 2013). One potential moderating factor is that some clinical studies were conducted exclusively with female volunteers, which could influence the generalizability of findings.

Contribution of CRF-2 receptors to stress-induced drug seeking is unclear, due to mixed methods and findings in the literature (Blacktop et al., 2011; Bruijnzeel et al., 2009; Wang et al., 2007; Zorrilla et al., 2014). CRF-2 receptors mediate anxiolysis (Bale et al., 2000; Kishimoto et al., 2000; Risbrough et al., 2004), suggesting CRF-2 agonists (not antagonists) are more appropriate anti-stress medication candidates but, presently, there are no published studies on this topic. Currently, there are no viable CRF-1 or CRF-2 receptor PET ligands for use in studies.

Brown et al. (2009) found that ICV infusion of NA reinstated cocaine seeking, which was blocked by pretreatment with the non-selective CRF antagonist D-Phe CRF_12-41_. In contrast, ICV CRF reinstated cocaine seeking but this was not blocked by clonidine pretreatment. Pretreating with the CRF antagonist or clonidine failed to block YOH-induced reinstatement. These findings suggest a functional interaction between NA and CRF systems in mediating stress-reinstatement of cocaine seeking, such that CRF receptor activation occurs downstream from NA sites of action (i.e., at extra-hypothalamic sites, independent of the HPA axis).

McReynolds et al. (2014) demonstrated that reinstatement of cocaine CPP using forced swim or systemic injection of the β2-receptor agonist clenbuterol was blocked by antalarmin or the β2-antagonist ICI-118,551; whereas clenbuterol reinstatement of cocaine CPP was only blocked by antalarmin but not the β2-antagonist. The authors hypothesized that stress-induced liberation of NA activates, via β2 receptors, CRF neurons that lead to drug-motivated responding.

#### 2.2.2 Glucocorticoids

Several early studies demonstrated that footshock stress, while increasing corticosterone (CORT) levels in rats, did not reinstate responding for heroin (Shaham et al., 1997), cocaine (Erb et al., 1998), or alcohol (Lê et al., 2000). Although acute pretreatment with the corticosteroid synthesis inhibitor, metyrapone, itself reinstated heroin-seeking behavior, neither acute nor chronic metyrapone exposure, nor adrenalectomy, attenuated footshock-reinstatement of heroin seeking (Shaham et al., 1997). These data implied that CORT does not specifically mediate reinstatement. Although one study found that acute pretreatment with the corticosteroid synthesis inhibitor, ketoconazole, blocked footshock reinstatement of cocaine seeking (Mantsch and Goeders, 1999), ketoconazole has other pharmacological actions, making it unclear whether this effect was HPA axis-specific. Furthermore, sustained ketoconazole treatment in a clinical study led to increased use of cocaine and heroin among methadone-maintained patients (Kosten et al., 2002).

Despite the important role of the HPA axis in addiction (Goeders, 2003), preclinical studies have found that glucocorticoid manipulations, rather than directly influencing drug seeking (given the negative findings above), may instead sensitize the animal to respond more in the presence of drug-related stimuli. Graf et al. (2013) found that exposing rats to footshock failed to reinstate cocaine seeking, but footshock did facilitate reinstatement to a subthreshold cocaine priming dose. Further, CORT administered to adrenalectomized rats also reinstated cocaine seeking with a subthreshold cocaine priming dose. Interestingly, CORT infused into the nucleus accumbens (NAc) was found to decrease DA clearance, suggesting CORT potentiation of NAc DA signaling may partly underlie stress-induced reinstatement. This research group also found that acute CORT alone did not reinstate extinguished cocaine CPP (McReynolds et al., 2017b) or self-administration (McReynolds et al., 2017a); however, CORT enhanced efficacy of a sub-threshold cocaine priming dose to reinstate cocaine CPP and self-administration (McReynolds et al., 2017a, b); thus, the stressor (CORT) “set the stage” for acute cocaine exposure to reinvigorate drug- or drug-paired responding. Also, these investigators found CORT and footshock-stress effects on cocaine-primed reinstatement of self-administration that were mediated by eCB signaling via CB_1_ receptors and the enzyme monoacylglycerol (McReynolds et al., 2016, 2017a). This research illustrates an interaction between the HPA axis and eCB system in the control of stress-occasioned relapse-like behaviors and suggests a role for using eCB modulators to attenuate the direct and indirect effects of stressors on drug-maintained behaviors (see section 2.6).

**Fig. 2:**
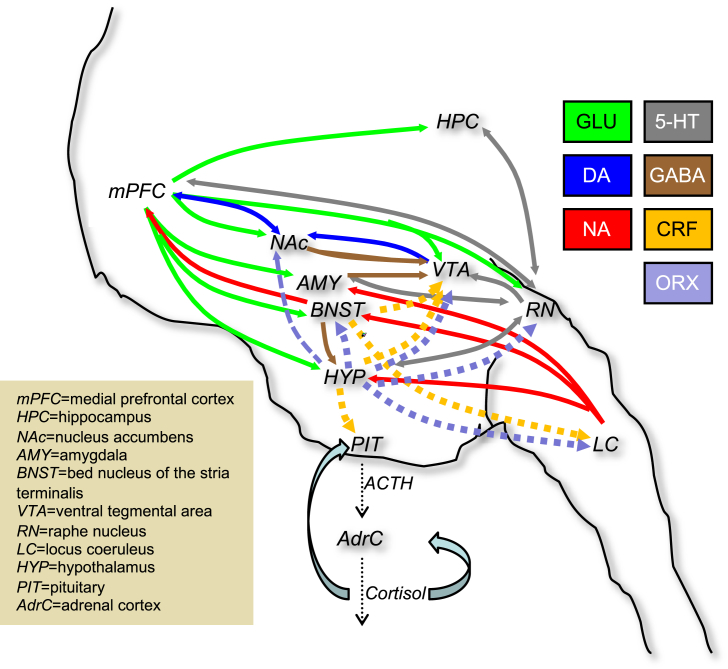
Systems Biology: Illustration of major neurochemical pathways that integrate and underlie stress-potentiated drug-seeking/use behaviors. See text for discussion of some of these inter-connections, as well as Table 3 relating NA function to other neurochemical systems.

Given the complexities of the HPA axis (e.g., negative feedback loops within the HPA axis; see Fig. 2), and the findings above that adrenalectomy or inhibition of corticosteroid synthesis did not block drug seeking, the potential utility of glucocorticoid receptor (GR) antagonists as anti-stress therapeutics must be seriously questioned. Also, nicotine abstinence is associated with HPA axis hypo-sensitivity (al’Absi et al., 2005; Semba et al., 2004), so it is unclear whether GR antagonists would be useful. On the other hand, a recent study found that the dual GR/progesterone antagonist mifepristone and the GR-specific antagonist CORT113176 each dose-dependently reduced escalated alcohol intake in rats and, in a companion clinical study, maintenance on mifepristone 600 mg/day (vs. placebo) reduced alcohol drinking among individuals with alcohol use disorder (Vendruscolo et al., 2015). Further investigation of these agents is warranted to shed light on the specific mechanisms through which this effect may occur, and whether it may be unique to alcohol or apply to other abused drugs.

Consistent with the systems biology focus in this review, NA and glucocorticoid systems produce co-operative effects in multiple paradigms (Dunn and Swiergiel, 2008; Forray and Gysling, 2004; Koob, 2008; Schwabe et al., 2010, 2012; Torregrossa et al., 2011; Valentino and Van Bockstaele, 2008; van Stegeren et al., 2010; Vasa et al., 2009). This prompted our recent study to co-activate these systems using separate and combined challenges with YOH and hydrocortisone (synthetic CORT) (Greenwald et al., in preparation). In a within-subject randomized crossover design, heroin-dependent buprenorphine-maintained volunteers were pretreated with oral YOH (0-, 27- and 54-mg) and CORT (0-, 20- and 40-mg) alone and combined. YOH/CORT increased opioid (hydromorphone) choices, relative to placebo pretreatment, while reducing money choices (i.e., stress increased opioid preference) but this effect was modulated by individual's pre-experimental variation in daily nicotine use. This pharmacological stress model was recently applied to cigarette smokers. Relative to placebo, YOH-54mg/CORT-10mg pretreatment induced similar physiological stress responses as above. Controlling for nicotine-dependence level, acute stress increased nicotine seeking (Woodcock et al., under review).

### 2.3 Serotonin system

Several studies implicate 5-HT neurotransmission in stress-induced reinstatement of alcohol seeking (whereas similar studies with abused drugs are lacking). In an initial study, alcohol seeking was reinstated by footshock and reversed by the 5-HT reuptake inhibitor fluoxetine (Lê et al., 1999). Subsequently, the 5-HT-1A agonist 8-OH-DPAT (which decreases 5-HT cell firing and transmitter release) infused in the median raphe nucleus (MRN) reinstated alcohol seeking; furthermore, intra-MRN infusion of CRF reinstated alcohol seeking (Lê et al., 2002). In a follow-up study, CRF antagonist infusion in the MRN attenuated YOH-reinstatement of alcohol seeking (Lê et al., 2013). Together, these findings point to 5-HT/CRF interaction within the MRN as mediating this stress effect. In addition to its primary α2-antagonist action, YOH also acts as a 5HT-1A partial agonist, which could partly explain its stress-reinstating efficacy. Furthermore, intra-MRN infusion of muscimol (GABA-A receptor agonist) reinstated alcohol seeking, implicating a 5-HT/GABA-A interaction in the MRN (Lê et al., 2008).

The 5-HT_3_ receptor antagonists, ondansetron and tropisetron (FDA-approved for treating nausea and vomiting), have been demonstrated to attenuate footshock-reinstatement of alcohol seeking in rats (Lê et al., 2006). However, these anti-stress effects have not yet been explored for other abused substances.

In summary, limited data suggest agents that promote 5-HT release, prevent reuptake, or block 5-HT-3 receptors could be useful therapeutics, although such effects may depend on raphe-mediated interactions; thus, systemic delivery could produce mixed effects. Given widespread clinical use of SSRIs and 5-HT-3 antagonists, systematic research across classes of abused drugs is warranted. Target-selective PET tracers are available that measure 5-HT transporter occupancy using [^11^C]DASB (for review, Spies et al., 2015); 5-HT synthesis using α-[^11^C]-methyl-L-tryptophan (Kumar et al., 2011); and 5-HT1A receptor binding using [*carbonyl*-^11^C]-WAY100635 or possibly [^11^C]-CUMI-101, but none for 5-HT-3 receptors (for review, Kumar and Mann, 2014).

### 2.4 Opioid system

Although the non-selective opioid receptor antagonist naltrexone (NTX) is used to treat alcohol and opioid use disorders, NTX does not block stress-induced alcohol or opioid use (e.g., Hyman et al., 2007; Lê et al., 1999). Notably, NTX can increase YOH reactivity (Rosen et al., 1999), and a clinical trial found that adding the α2-agonist guanfacine to NTX did not improve opioid abstinence over NTX alone (Krupitsky et al., 2013).

It is presently unclear whether opioid agonist medications might (in addition to their withdrawal-suppressing and opioid blockade properties; see Greenwald et al., 2014) attenuate stress-reactivity among individuals with opioid use disorder. One animal study found that maintenance on the *mu*-agonist methadone (MTD) did not block stress-reinstatement of heroin seeking (Leri et al., 2004). Results from cross-sectional clinical studies suggest MTD might blunt stress-reactivity (Kakko et al., 2008; Kreek et al., 1984; Schluger et al., 2001, 2003) but those studies have methodological flaws, e.g., variable maintenance doses, potential pre-morbid differences between patients and controls, and failure to match controls on other substance use. Buprenorphine (BUP) is a partial *mu*-receptor agonist/*kappa*-receptor antagonist with rapid-onset antidepressant and anxiolytic effects in animal models (Falcon et al., 2015) and human studies (Bershad et al., 2015; Bodkin et al., 1995; Karp et al., 2014; Nyhuis et al., 2008). In the clinical dosing range for opioid use disorder, BUP may be stress-protective *via* one or both of these mechanisms; our lab is presently investigating this issue.

#### 2.4.1 Kappa-opioid receptor (KOR) antagonists

Stressors have been shown to release dynorphin and activate *KOR*s located on DA and 5-HT neurons, thereby reducing DA and 5-HT levels (Carlezon et al., 2006; Ebner et al., 2010; Gehrke et al., 2008; Land et al., 2008; Zhang et al., 2007) that, in turn, are associated with negative-hedonic effects of stressors (Fig. 1). Furthermore, *KOR* agonist administration reliably reinstates responding for cocaine (see Table 1).

Accordingly, *KOR* antagonists are being evaluated as potential anti-depressants (Carr et al., 2010; Filho et al., 2013; Grimwood et al., 2011; Zhang et al., 2007) and anti-stress/drug-relapse prevention agents. As summarized in Table 2, *KOR* antagonists reliably attenuate stress-reinstated responding for nicotine, alcohol (but see Schank et al., 2012), cocaine, and heroin. Although no tests of *KOR* antagonism of stress-related substance abuse have been conducted in humans to date, PET ligands for measuring *KOR* occupancy are available including [^11^C]-LY2795050 (Naganawa et al., 2014) and [^18^F]-LY2459989 (Li et al., 2018).

Evidence also suggests dynorphin projections to NA and GABA neurons in the locus coeruleus (Kreibich et al., 2008; Reyes et al., 2007) modulate stress-related drug reinforcement. Studies in rats have identified *KOR*/noradrenergic interactions in the locus coeruleus that mediated reinstatement of cocaine lever-responding (Al-Hasani et al., 2013b), and *KOR*/serotonergic projections from the dorsal raphe to the NAc that mediated reinstatement of cocaine conditioned place preference (CPP) (Land et al., 2009). Finally, interactions between dynorphin/*KOR*s and CRF have been proposed as one mechanism by which stressors lead to changes in allostatic load, altering the motivational valence of the abused substance (Bruchas et al., 2010; Koob, 2015).

#### 2.4.2 Delta-opioid receptor (DOR) compounds

*DOR*s are a potential anti-stress target, given their established role in anxiety-like behaviors in animal models; however, their pharmacology is incompletely understood, including differing functions of *DOR* subtypes, stress-induced translocation of *DOR*s from cytoplasm to cell surface, and potential for *DOR*s to form heteromers with *mu*-receptors (van Rijn et al., 2013). In the only relevant study to date, YOH-reinstatement of alcohol seeking was attenuated by a *DOR-1* antagonist, SoRI-9409 (Nielsen et al., 2012). Further research with *DOR* subtype-specific agonist and antagonists is needed to determine whether such agents could be useful anti-stress therapeutics. The PET ligand [^11^C]methyl-naltrindole is clinically available (Madar et al., 1996; Smith et al., 1999) and has been used to measure *DOR* binding potential in alcohol-dependent individuals (Wand et al., 2013; Weerts et al., 2008, 2011).

### 2.5 Nociceptin receptor (NOP) compounds

Although the NOP system is involved in stress-reactivity and addictive behaviors, historically less clear is whether NOP agonists or antagonists should be effective anti-stress agents. On the one hand, NOP agonists have shown therapeutic promise against stress-reinstatement of alcohol seeking. Footshock-reinstatement of alcohol seeking was attenuated by ICV administration of nociceptin (Martin-Fardon et al., 2000) and systemic administration of the non-peptide, brain-penetrant NOP agonist MT-7716 in post-dependent rats but not alcohol-naïve rats (de Guglielmo et al., 2014). The latter findings suggest a history of alcohol dependence dysregulates the nociceptin system, which can be reversed with NOP agonist treatment. A different small-molecule NOP agonist, SR-8993, blocked YOH-reinstatement of alcohol seeking (Aziz et al., 2016). Conversely, other studies have found that NOP antagonists and NOP knockout mice produce anti-obesity and anti-depressant effects (review by Witkin et al., 2014), and the orally bioavailable, brain-penetrant, NOP antagonist LY2940094 blocked YOH-reinstatement of alcohol seeking in alcohol-preferring rats (Rorick-Kehn et al., 2016).

Witkin et al. (2014) proposed a hypothesis to reconcile these differences; specifically, nociceptin serves as part of a stress-coping negative-feedback mechanism, eliciting a stress-like response within the HPA axis but anti-stress effects at extra-hypothalamic sites. For future studies, the PET ligand [^11^C]NOP-1A (Lohith et al., 2012) is available to measure NOP binding potential in human subjects (Narendran et al., 2017).

### 2.6 Endocannabinoid (eCB) system

Initial studies of the eCB system as an anti-stress target focused on CB_1_ receptor antagonists. Administration of the CB_1_ antagonist rimonabant to rats failed to attenuate footshock-reinstatement of alcohol seeking (Economidou et al., 2006; 2007) and cocaine seeking (De Vries et al., 2001). Moreover, side effects of rimonabant in clinical trials led to discontinuation of its therapeutic development. The neutral CB_1_ antagonist AM4113 dose-dependently reduced YOH-reinstatement of nicotine seeking without altering inactive-lever or food-maintained responding acutely, although chronic treatment led to weight loss (Gueye et al., 2016). In contrast, the CB_1_ antagonist AM251 blocked forced swim-induced cocaine reinstatement (Vaughn et al., 2010) and AM251 blocked CRF- but not footshock-reinstatement of cocaine seeking (Kupferschmidt et al., 2012). Given these mixed effects of CB_1_ antagonists on stress-reinstatement, it is unclear whether findings may depend on interactions between the CB_1_ antagonist, stressor, and abused substance.

An alternative strategy is to modulate signaling indirectly within the eCB system. One promising approach is to promote eCB transmission by facilitating actions of the endogenous ligand anandamide (AEA). Although acute administration of the FAAH inhibitor URB597 (which increases AEA levels by inhibiting its catabolism) did not attenuate footshock- or YOH-reinstatement of alcohol seeking (Cippitelli et al., 2008), Chauvet et al. (2014) argued that chronic administration of anti-stress medication is a preferable model, because (1) this directly pertains to the human condition, (2) chronic exposure controls for possible tolerance development, and (3) medication-induced neuroadaptations may be required for a therapeutic effect. Using this approach, they demonstrated that URB597 significantly attenuated YOH-reinstatement of cocaine seeking (Chauvet et al., 2014). Targeting fatty acid binding proteins, which transport AEA intracellularly to FAAH for degradation, is a related strategy to interfere with stress-induced drug seeking (Hamilton et al., 2018).

The eCB cannabidiol (CBD) has diverse neuropharmacological actions but salient among these includes enhancing AEA levels with weak antagonist actions at CB_1_ and CB_2_ receptors (Campos et al., 2012; Izzo et al., 2009; Kathman et al., 2006; McPartland et al., 2015; Mijangos-Moreno et al., 2014; Pertwee, 2008; Petitet et al., 1998; Russo et al., 2005; Thomas et al., 2007). Transdermal CBD administration was recently found to attenuate acute and repeated YOH-reinstatement of cocaine and alcohol seeking and produced anxiolytic and anti-impulsive effects without disrupting other reward behavior or locomotion, and this profile of effects endured long after CBD was biologically detectable (Gonzalez-Cuevas et al., 2018). Further studies are needed, but CBD represents a novel anti-stress therapeutic approach.

PET radiotracers are clinically available that measure binding potential in the eCB system including [^11^C]-CURB (Boileau et al., 2015; Rusjan et al., 2013) and [^11^C]-MK3168 (Postnov et al., 2018) for FAAH; [^11^C]-OMAR (Normandin et al., 2015) and [^11^C]-MePPEP and [^18^F]-FMPEP-d (Terry et al., 2010) for CB_1_ receptors; and [^11^C]-NE40 for CB_2_ receptors (Ahmad et al., 2013). Measurement of eCB levels in biological matrices is promising and undergoing refinement (Dlugos et al., 2012; Feuerecker et al., 2012; Vaughn et al., 2010; Walter et al., 2013; Witkamp and Balvers, 2016).

### 2.7 Orexin system

Orexin fibers (originating mostly in the lateral hypothalamus) project widely in the brain, including to VTA, NAc and extended amygdala and, thus, are well-positioned to influence motivated behavior. The orexin system has been demonstrated to influence stress-induced seeking for several abused substances. ICV administration of orexin-1 but not orexin-2 peptide reinstates drug seeking (Boutrel et al., 2005; Harris et al., 2005; Plaza-Zabala et al., 2010; Wang et al., 2009), consistent with a broader role for orexin-1 in compulsive behavior (Pich and Melotto, 2014). Notably, the orexin-1 receptor-selective antagonist SB-334867 dose-dependently reduced footshock- and YOH-reinstatement of cocaine responding, particularly in rats given long-access to cocaine, a model of escalated drug use (Boutrel et al., 2005; Schmeichel et al., 2017; Wang et al., 2009). A set of studies found that restraint stress initially activated hypothalamic orexin neurons to release orexins into the VTA, activating postsynaptic VTA DA neurons, causing retrograde eCB inhibition of GABA release, leading to VTA DA disinhibition that resulted in reinstatement of cocaine CPP (Tung et al., 2016).

Systemic pretreatment with SB-334867 attenuated YOH-reinstatement of alcohol seeking, without altering locomotion (Richards et al., 2008). However, a later study found that only micro-injection of an orexin-2 receptor antagonist (but not SB-334867) into the pontine *nucleus incertus* attenuated YOH-reinstatement of alcohol seeking (Kastman et al., 2016). These mixed results could be due to different methods. A human study found that plasma concentrations of orexin – which may reflect diffusion of the peptide between CNS and peripheral compartments (Kastin and Akerstrom, 1999) – during alcohol abstinence positively correlated with psychological distress levels (von der Goltz et al., 2011); thus, it is plausible that orexin modulates stress- or abstinence-related alcohol use. However, there are also contrary data in cigarette smokers, showing a negative relationship between peripheral orexin-1 levels and nicotine craving (von der Goltz et al., 2010).

For nicotine, SB-334867 blocked reinstating effects of ICV orexin and footshock on nicotine seeking; in contrast, SB-334867 did not block CRF-induced nicotine reinstatement, and the CRF-1 antagonist antalarmin did not block ICV orexin-induced nicotine reinstatement, indicating autonomous control of stress-related drug-seeking across these two systems (Plaza-Zabala et al., 2010).

For opioids, pretreatment with either orexin-1 or orexin-2 receptor antagonists in the NAc shell (which processes positive and negative affectively-valenced behaviors) attenuated reinstatement of morphine CPP (Qi et al., 2013).

Suvorexant is a non-selective orexin-1/2 receptor antagonist approved for treating insomnia. Evidence indicates that orexin-2 antagonism is responsible for the sleep-inducing effect (Andrews et al., 2016). Consistent with findings above, orexin-1 antagonists such as GSK1059865 (Gozzi et al., 2011) are being developed as anti-stress and anti-addiction pharmacological tools, although none are clinically available at this time. Although there are emerging orexin-2 ligands for PET imaging such as [^11^C]-MK-1064 (Gao et al., 2016) and [^11^C]-CW4 (Wang et al., 2013), at this time there are no viable orexin-1 radiotracers.

### 2.8 Glutamatergic system

Multiple signals converge on glutamate signaling to alter stress-induced drug seeking. The effect of footshock stress on cocaine responding was shown to depend on glutamate via dorsal PFC/NAc core interaction (McFarland et al., 2004) and VTA CRF signaling (Wang et al., 2005; Williams et al., 2014), ultimately leading to increase NAc DA transmission (Wise, 2009). Cold-water forced-swim stress was shown to alter AMPA/NMDA receptor ratio in the NAc shell, an effect that was reversed with the glucocorticoid receptor antagonist RU486 (Campioni et al., 2009). NA signaling through α2 and β2 receptors in the dorsal BNST, can bidirectionally modulate glutamate transmission (Egli et al., 2005). Although these examples are not exhaustive, they illustrate that glutamate signaling in stress-related behavior is mostly secondary to other systems. This implies that a direct pharmacological approach to modulating glutamate function may be challenging for treating stress-related substance use.

Two agents with glutamate transmission-dampening properties, a mGluR2/3 agonist (LY379268) and mGluR5 antagonist (MTEP), each dose-dependently reduced footshock-reinstatement of cocaine seeking (Martin-Fardon and Weiss, 2012) and alcohol seeking (Sidhpura et al., 2010; Zhao et al., 2006). These findings suggest mGluR2/3 and mGluR5 targets (primarily located in pre-/peri- and post-synaptic spaces, respectively) should be studied further, given the established anxiolytic effects of LY379268 and MTEP in animal models (e.g., Kenny and Markou, 2004).

On the other hand, a review of medications for treating cocaine addiction (not specific to stress-induced drug seeking) suggested that allosteric modulators could be pursued (Kalivas and Volkow, 2011). Given the evidence above, reasonable candidates to be considered would be positive allosteric modulators of mGluR2/3 receptors and negative allosteric modulators of mGluR5 receptors. Given similar neuroadaptations from chronic alcohol (relative to cocaine) use, including a hypersensitive glutamate system (Vengeliene et al., 2008), it may be fruitful to investigate these allosteric modulators for stress-induced alcohol use. Yet, an initial test of this hypothesis found that the mGluR2-selective positive allosteric modulator AZD8529 attenuated cue- but not footshock-reinstatement of alcohol seeking in rats (Augier et al., 2016). Those authors note that this difference in findings across studies could imply that mGluR3 rather than mGluR2 receptors are distinctly involved in stress-related drug responding, or that orthosteric agonists and positive allosteric modulators could modulate glutamate function in distinct ways.

Several biomarkers are available to measure glutamate function in human subjects. Emerging PET radiotracers for measurement of brain glutamate targets include [^18^F]-FIMX for mGluR1 receptors (Zanotti-Fregonara et al., 2016), and [^11^C]-ABP688 (Kågedal et al., 2013) and [^18^F]-FPEB (Sullivan et al., 2013) for mGluR5 receptors. High-field proton magnetic resonance spectroscopy (^1^H-MRS) has proved sensitive to pharmacotherapy-related repeated-measures change in brain-regional glutamate concentrations in substance abusers (Greenwald et al., 2015; Umhau et al., 2010).

### 2.9 GABAergic system

The GABA-A agonist muscimol, when injected into the median raphe nucleus, reinstated alcohol seeking (Lê et al., 2008). However, intra-VTA modulation of GABA-A receptors using the GABA-A antagonist bicuculline did not block footshock- or intra-VTA CRF-induced reinstatement of cocaine seeking (Blacktop et al., 2016). However, it is possible that a GABA-A positive allosteric modulator could offer a useful alternative; one such compound is in development (Martinez Botella et al., 2017).

In contrast, the GABA-B agonist baclofen was found to block YOH-induced alcohol seeking (Williams et al., 2016) and to attenuate forced swim stress-reinstatement of morphine CPP (Meng et al., 2014). However, these encouraging results must be reconciled with conflicting findings that intra-VTA infusion of a GABA-B antagonist, 2-hydroxysaclofen, blocked reinstatement of cocaine seeking by footshock and intra-VTA CRF administration (Blacktop et al., 2016). Thus, it is unclear whether using a GABA-B agonist or antagonist would be preferable against stress-induced drug seeking, or whether this might vary by the abused substance or route of delivery.

GABA modulation with pregabalin (a clinically available medication) was found to block YOH-reinstatement of alcohol seeking (Stopponi et al., 2012) and cocaine seeking (de Guglielmo et al., 2013). The GAT-1 inhibitor tiagabine has been demonstrated to reduce anxiety- and depressive-like behaviors in animal models (Thoeringer et al., 2010) but has not yet been tested in experimental models of stress-induced drug seeking/use.

Biomarkers for GABA system targets include the PET radiotracer [^11^C]-flumazenil for GABA-A receptors (Jucaite et al., 2017; Lingford-Hughes et al., 2005), and ^1^H MRS imaging for cortical GABA concentrations (Mason et al., 2006; Streeter et al., 2005).

### 2.10 Other candidates

For completeness of coverage, this section briefly notes promising alternative candidates with relatively less empirical evidence.

#### 2.10.1 Neuropeptides

Oxytocin is synthesized in the hypothalamus and binds to oxytocin and vasopressin receptors in brain regions implicated in regulating stress-reactivity, emotional and social behaviors (Heinrichs and Domes, 2008; Neumann and Landgraf, 2012; Windle et al., 2004). Studies of oxytocin suggest its clinical potential for several psychiatric conditions (Keech et al., 2018; Meyer-Lindenberg et al., 2011; Naja and Aoun, 2017; Quintana et al., 2017) although some therapeutic effects are likely to be sex-dependent (Bisagno and Cadet, 2014). Overall, few studies relate to substance abuse, and oxytocin might have greater promise for treating stress-related psychiatric conditions comorbid with SUDs (Zanos et al., 2017). An oxytocin analogue, carbetocin, attenuated forced-swim stress-reinstatement of morphine CPP, while attenuating stress-induced plasma CORT response (Zanos et al., 2014). Amygdala arginine vasopressin (AVP)1b receptors have been implicated in footshock-reinstatement of heroin seeking (Zhou et al., 2008, 2015), suggesting V1b receptor antagonism might be a viable target for blocking stress-related relapse. One V1b antagonist (ABT-436) is in clinical trials for treatment of alcohol use disorder (Ryan et al., 2017).

Neuropeptide S (NPS) acts at the NPS receptor to increase arousal and locomotion while decreasing anxiety-like behavior (Leonard et al., 2008; Rizzi et al., 2008; Xu et al., 2004). This paradoxical profile – anxiolysis but increased locomotion (often associated with reinforcing effects) – resembles that of nicotine, creating uncertainty as to whether agonists or antagonists of this system would be optimal anti-stress candidates. Schmoutz et al. (2012) adopted an NPS receptor antagonist approach, based on the idea that NPS may promote addictive behaviors. Using RTI-118, they found it decreased YOH- as well as cue- and cocaine priming-induced reinstatement of cocaine seeking, indicating a broad efficacy profile (Schmoutz et al., 2012). NPS receptors are co-localized with other hypothalamic-originating neuropeptides discussed in this review, including orexin and CRF receptors. Hypothalamic NPS infusion has been shown to reinstate alcohol and cocaine seeking, effects that are reversed by the orexin-1 antagonist SB-334867 (Cannella et al., 2009; Kallupi et al., 2010; Ubaldi et al., 2016) and the CRF-1 antagonist antalarmin (Paneda et al., 2009).

Neuropeptide Y binds multiple receptor subtypes; however, most studies have investigated Y1 (mostly postsynaptic) and Y2 (mostly presynaptic) receptors with the latter negatively modulating NPY release (Colmers et al., 1991; Eva et al., 2006; King et al., 1999) to produce numerous biobehavioral effects including anxiolysis (Heilig, 2004). The actions of NPY counteract CRF-induced anxiogenesis (Heilig et al., 1994; Sajdyk et al., 2004; Valdez and Koob, 2004). Central NPY infusion blocked YOH-reinstatement of alcohol seeking (Cippitelli et al., 2010). Systemic pretreatment with the Y2-selective antagonist, JNJ-31020028, failed to block footshock-reinstatement of alcohol responding (Cippitelli et al., 2011). A similar stress-reinstatement blocking test has not been reported for a Y1-selective agonist. Experimental results from studies of this system could be complicated by findings that Y1 and Y2 receptors (which have opposite functions regarding anxiety-like behavior) can form homo- and hetero-dimers whose net effect might negate the effects of receptor-selective stimulation (Dinger et al., 2003; Silva et al., 2003).

Substance P is a tachykinin-family neuropeptide selective for the neurokinin-1 (NK-1) receptor. The NK-1 receptor antagonist aprepitant is FDA-approved for treating chemotherapy-induced nausea and biosimilars are in the pipeline. Clinical development of NK-1 antagonists has met with difficulties perhaps, not surprisingly, because these agents share functional features with CRF-1 antagonists that have associated negative findings (see section 2.2.1; Schank and Heilig, 2017). Preclinical studies have shown that the NK-1 antagonist L822429 blocks YOH- or footshock-reinstatement of alcohol and cocaine seeking (Schank et al., 2011, 2014, 2015). Although a phase 2 clinical study found that the NK-1 antagonist LY686017 decreased spontaneous and psychological stress-induced alcohol craving and cortisol responses in high trait-anxious, alcohol-dependent volunteers (George et al., 2008), a subsequent placebo-controlled study of aprepitant administered to alcohol-dependent patients with co-occurring PTSD failed to replicate these findings (Kwako et al., 2015a). Although it remains unclear whether NK-1 antagonists could reduce acute stress-induced effects among individuals who are not highly anxious, this would considerably restrict the clinical utility of this class of medication. The PET ligand [11C]-GR205171 is available for measuring NK-1 binding potential in humans (Frick et al., 2015; Ridler et al., 2014; Spinelli et al., 2014).

Relaxin-3 is a peptide acting at its cognate receptor RXFP3 and, similar to orexin, relaxin-3 agonists increase stress-reactivity, food intake and arousal. In contrast, relaxin-3 antagonism, at least partly mediated via the BNST, blocked YOH-reinstatement of alcohol- but not sucrose-seeking (Ryan et al., 2013); furthermore, RXFP3 deletion blocked physical (repeated restraint followed by swim) stress-reinstatement of alcohol seeking without disrupting baseline alcohol or saccharin intake, or hepatic metabolism of alcohol (Walker et al., 2015). Thus, relaxin-3 antagonists may be reasonable anti-stress agents, although efficacy with other abused drugs has not been tested to date.

#### 2.10.2 Non-peptides

FDA-approved nicotine replacement treatments (NRTs) do not attenuate stress-induced drug seeking or use (Kotlyar et al., 2006; Ray et al., 2013). Interestingly, the α3β4 nAChR partial agonist AT-1001 dose-dependently attenuated YOH-reinstatement of nicotine seeking (Yuan et al., 2017). Furthermore, AT-1001 selectively blocked YOH- but not cue-reinstatement of alcohol seeking without affecting baseline alcohol or food intake (Cippitelli et al., 2015). This novel approach could potentially address some limitation of current NRTs and assist the many individuals who concurrently use tobacco and alcohol.

Pioglitazone, agonist at the peroxisome proliferator-activated receptor-gamma (PPARγ) subtype, was shown to attenuate YOH-reinstatement of alcohol seeking (Stopponi et al., 2011, 2013) and heroin seeking (de Guglielmo et al., 2017).

## 3 Discussion

The development of anti-stress medications is critical for the advancement of treating all SUDs. The impact of stressors extends across abused substances (which contrasts with the effects of drug-priming and drug-cue exposure, which tend to be specific to drug classes). Therefore, progress in this field has significant potential to apply scientifically across all drug classes and to improve treatment of all patients with SUDs. This review describes available evidence on promising neuropharmacological approaches, using mechanistic studies based on chemical and non-chemical probes (Table 1). Although studies of Δ^9^-THC/marijuana were surveyed for this review, there is a paucity of studies in this area.

Based on the quality of evidence to date, promising first-tier neurochemical targets include: NA (α1-and β-antagonist, α2 agonist), *kappa*-opioid antagonist, NOP antagonist, orexin-1 antagonist, and eCB modulation (e.g., cannabidiol, FAAH inhibition); second-tier candidates may include CRF-1 antagonists, serotonergic agents (reuptake inhibitors, 5-HT-3 antagonists), glutamatergic agents (mGluR2/3 agonist/positive allosteric modulator, mGluR5 antagonist/negative allosteric modulator), GABA-signaling promoters (e.g., pregabalin, tiagabine), vasopressin 1b antagonist, NK-1 antagonist, and PPAR-γ agonist (e.g., pioglitazone). DA antagonists were excluded from this review because, although brain site-specific studies in animals support a role for DA in mediating stress-induced drug seeking (McFarland et al., 2004), when DA antagonists are administered systemically (and especially chronically) they produce side effects that do not translate well into clinical practice.

Table 2 complements this review by adding a layer of theoretical analysis pertaining to affective/motivational mechanisms that may be modulated while intervening in these neurochemical systems. I propose the overarching hypothesis that, to be effective, anti-stress medications for substance use disorders must alleviate the multidimensional burden of stressors that can lead to behavioral deficits including avoidance (valence dimension), hyperactivation (arousal dimension), and impulsivity (control dimension), that can perpetuate substance use and its adverse consequences. Table 2 illustrates that few candidate pharmacotherapeutic approaches are likely to tackle all these problems. Accordingly, to address these affective/motivational mechanisms of stress-related substance use, it seems advisable to combine agents with actions at complementary targets for greater efficacy; however, it should be noted that systematic studies are lacking except for interactions with the NA system (Table 3). Future research could be directed at whether some agents may function to desensitize subjects from chronic stress-induced effects (e.g., glucocorticoid receptor antagonist), whereas other agents might help to block acute stress-induced effects (e.g., α2 agonist or α1 antagonist). It is plausible that both types of agents together may be more effective than either alone. Some agents may also have additional effects beyond anti-stress, such as to block cue-induced drug seeking (e.g., eCB modulators, orexin-1 antagonist).

A key methodological factor concerns whether medication development studies employ acute vs. chronic administration. Although acute medication administration is the dominant paradigm to date, partly due to its efficiency, Chauvet et al. (2014) effectively argued that chronic exposure is desirable for applied clinical relevance, to avoid false negative results attributable to development of tolerance, and to enable within- and/or between-system neuroadaptations (including epigenetic changes) to occur. Future studies should carefully weigh these issues, e.g., whether to screen medications acutely (but perhaps also chronically) during early-stage development but move to chronic dosing in phase 2 studies.

### 3.1 Biomarkers

Programmatic studies should include biomarkers of stress-reactivity and medication targeting to confirm mediation of effects on drug-seeking behavior. Section 2 mentioned clinically available PET ligands for measuring occupancy of molecular targets and use of ^1^H-MRS for measuring glutamate and GABA brain-regional concentrations. These CNS biomarkers should be paired with measurement of medication plasma concentrations, and target occupation/stimulation and plasma pharmacokinetic data should be correlated with one another and with pharmacodynamic effects (clinical endpoints) to generate exposure-response functions. We successfully applied a similar PK/PD modeling strategy for optimizing sublingual and depot buprenorphine treatment of opioid use disorder (Greenwald et al., 2014; Nasser et al., 2014, 2016) and this approach was recently applied in the development of the NOP antagonist LY2940094 (Rorick-Kehn et al., 2014, 2016).

### 3.2 Heterogeneity of effect

As indicated in Table 2, which maps neurobiological and motivational foundations of anti-stress medications, it seems unlikely that any single medication will be highly effective for attenuating stress-mediated negative-hedonic, arousal/activating, and disinhibiting/loss-of-control effects on drug-maintained behaviors. Furthermore, chronic dosing may lead to between-system adaptations resulting in loss of efficacy over time, so it may be advantageous to employ a redundant approach (e.g., use two medications that each target one motivational dimension, but via different pharmacological stimulation). This approach emphasizes using one or more anti-stress medications that exert actions that address multiple motivational features of stress-reactive drug use. Using the model proposed here, a predictable consequence of deploying highly targeted compounds would be restricted efficacy. Individual difference factors need to be considered in the context of medication development; inattention to these variables will also limit efficacy (section 1.4).

### 3.3 Summary

This review has identified promising, evidence-based neurochemical mechanisms and potential pharmacotherapeutic leads to combat stress-induced substance seeking/use, a major problem for all SUDs. Medications that prove safe and effective could be added to existing SUD pharmacotherapies (e.g., agonist therapies for opioid or nicotine use disorder) to augment treatment efficacy, as most FDA-approved agonist treatments were not designed as anti-stress agents. To address motivational dimensions of stress-related substance use, it will be theoretically and pragmatically valuable to test agents with complementary actions alone and in combination to improve efficacy. As these agents are developed, it will be important to recognize numerous clinically-relevant factors (some of which could be modeled in animal studies) that could mediate/moderate the efficacy of anti-stress agents. Finally, progress in developing anti-stress medications will also depend on use of reliable CNS biomarkers to validate exposure-response relationships.

## Author disclosures

### Role of funding source

NIH 2 R01 DA015462 from the National Institute on Drug Abuse, Helene Lycaki/Joe Young Sr. funds (State of Michigan), and the Detroit Wayne Mental Health Authority supported preparation of this manuscript.

### Contributors

The author is responsible for the content of this manuscript, but gratefully acknowledges the many valuable contributions offered by two anonymous reviewers.

### Conflicts of interest

The author has received compensation as a scientific consultant to Indivior, Inc., which manufactures and markets buprenorphine products, and he has co-authored publications without compensation regarding RBP-6000 (Sublocade™).
